# A large-scale and PCR-referenced vocal audio dataset for COVID-19

**DOI:** 10.1038/s41597-024-03492-w

**Published:** 2024-06-27

**Authors:** Jobie Budd, Kieran Baker, Emma Karoune, Harry Coppock, Selina Patel, Richard Payne, Ana Tendero Cañadas, Alexander Titcomb, David Hurley, Sabrina Egglestone, Lorraine Butler, Jonathon Mellor, George Nicholson, Ivan Kiskin, Vasiliki Koutra, Radka Jersakova, Rachel A. McKendry, Peter Diggle, Sylvia Richardson, Björn W. Schuller, Steven Gilmour, Davide Pigoli, Stephen Roberts, Josef Packham, Tracey Thornley, Chris Holmes

**Affiliations:** 1 London Centre for Nanotechnology, University College London, London, UK; 2 Division of Medicine, University College London, London, UK; 3 King’s College London, London, UK; 4 The Alan Turing Institute, London, UK; 5 Imperial College London, London, UK; 6 UK Health Security Agency, London, UK; 7 Institute of Health Informatics, University College London, London, UK; 8 Centre for Stress and Age-Related Disease, School of Applied Sciences, University of Brighton, Brighton, UK; 9 University of Oxford, Oxford, UK; 10 University of Surrey, Guildford, UK; 11 The Surrey Institute for People-Centred AI, Centre for Vision, Speech and Signal Processing, Guildford, UK; 12 University of Lancaster, Lancaster, UK; 13 CHI, MRI, Technical University of Munich, Munich, Germany; 14 University of Nottingham, Nottingham, UK

**Keywords:** Diagnostic markers, Respiratory signs and symptoms

## Abstract

The UK COVID-19 Vocal Audio Dataset is designed for the training and evaluation of machine learning models that classify SARS-CoV-2 infection status or associated respiratory symptoms using vocal audio. The UK Health Security Agency recruited voluntary participants through the national Test and Trace programme and the REACT-1 survey in England from March 2021 to March 2022, during dominant transmission of the Alpha and Delta SARS-CoV-2 variants and some Omicron variant sublineages. Audio recordings of volitional coughs, exhalations, and speech were collected in the ‘Speak up and help beat coronavirus’ digital survey alongside demographic, symptom and self-reported respiratory condition data. Digital survey submissions were linked to SARS-CoV-2 test results. The UK COVID-19 Vocal Audio Dataset represents the largest collection of SARS-CoV-2 PCR-referenced audio recordings to date. PCR results were linked to 70,565 of 72,999 participants and 24,105 of 25,706 positive cases. Respiratory symptoms were reported by 45.6% of participants. This dataset has additional potential uses for bioacoustics research, with 11.3% participants self-reporting asthma, and 27.2% with linked influenza PCR test results.

## Background & Summary

The scale and impact of the COVID-19 pandemic has created a need for rapid and affordable point-of-care diagnostics and screening tools for infection monitoring. The possibility of accurate and generalisable detection of COVID-19 from voice and respiratory sounds using audio classification on a smart device has been hypothesised as a way to provide a non-invasive, affordable and scalable option for COVID-19 screening for both personal and public health monitoring^1^. However, prior machine learning studies to determine the feasibility of COVID-19 detection from audio have largely relied on datasets which are too small or unrepresentative to produce a generalisable model, or which include self-reported COVID-19 status, rather than gold standard PCR (Polymerase Chain Reaction) testing for SARS-CoV-2 infection (see Table 1). These datasets have a relatively small proportion of positive cases, and include inadequate metadata for statistical evaluation. They largely do not enable studies using them to meet diagnostic reporting criteria (for example the STARD 2015^2^ and forthcoming STARD-AI^3^ criteria), such as: reporting the interval between reference test and recording, random sampling, or avoiding case control where positives and negatives are sourced from different recruitment channels.

**Table 1:** Summary of currently available COVID-19 biomedical acoustics datasets as of 2023-12-16.

Dataset	COVID- Positive/ Total Participants (% COVID Positive)	COVID Label (% PCR results)	Modalities	Testing interval reported?	Metadata
The UK COVID-19 Vocal Audio Dataset - Open Access Version^9^ (this study)	25,706/72,999 (35.2%)	PCR (96.7%) LAMP Lateral Flow	Cough, Exhalation	Yes	Symptoms; Respiratory Conditions; Age group; Gender; Smoker status; Ct values; Influenza status
Tos COVID-19^21^	25,664/139,986 (18.3%)	PCR (19.4%) Lateral Flow	Cough	Yes (±3 days)	Symptoms; Close Contact; Risk group; Test location
COVID-19 Sounds^31^	2,106*/36,116 (5.8%*)	Self-reported	Cough, Exhalation, Speech	Yes	Symptoms; Health conditions; Age; Sex; Smoker status; Language; Hospitalisation status
COUGHVID^18^	1,010/20,072 (5.3%)	Self-reported, Clinician annotation	Cough	No	Symptoms; Respiratory conditions; Age; Gender; Location
smarty4covid^42^	732/4,303 (17.0%)	Self-reported, Clinician annotation	Cough, Exhalation, Speech	Yes (3 days)	Symptoms; Health conditions; Vaccination Status; Hospitalisation status; Vital signs; Smoker status; Anxiety levels; Age; Gender; Body mass index
Covid19-Cough^43^	682/1,324 (51.5%)	PCR (28.9%), Self-Reported	Cough	No	Symptomatic
Coswara^44^	389/2,233 (17.4%)	Self-reported	Cough, Exhalation, Speech	No	Health conditions; Symptoms; Age; Gender; English proficiency; Country; Locality; State; Smoker status; Vaccination Status
Virufy^45^	143/456 (31.4%)	PCR (93.2%), Self reported	Cough	No	Symptoms; Medical conditions; Age; Sex; Smoker status

*COVID-positive samples, individual COVID-positive participants may have recorded a sample more than once.

Following the publication of initial studies reporting accurate classification of SARS-CoV-2 infection from vocal and respiratory audio^4,5^, the UK Health Security Agency (UKHSA, formerly NHS Test and Trace, the Joint Biosecurity Centre, and Public Health England) were commissioned to collect a dataset to allow for the independent evaluation of these studies. Dataset analysis was carried out by The Alan Turing Institute and Royal Statistical Society (Turing-RSS) Health Data Lab (https://www.turing.ac.uk/research/research-projects/turing-rss-health-data-lab). A dataset larger than the majority of existing datasets was needed to provide sufficient instances of various recording environments and mobile devices (information which is not collected), and to provide sufficient instances for the thousands of features or representations typically produced from short vocal audio samples^6^. Such a dataset also needed to be sufficiently large and diverse to validate model performance across various participant demographic groups and presentations of SARS-CoV-2 infection.

**Fig. 1:**
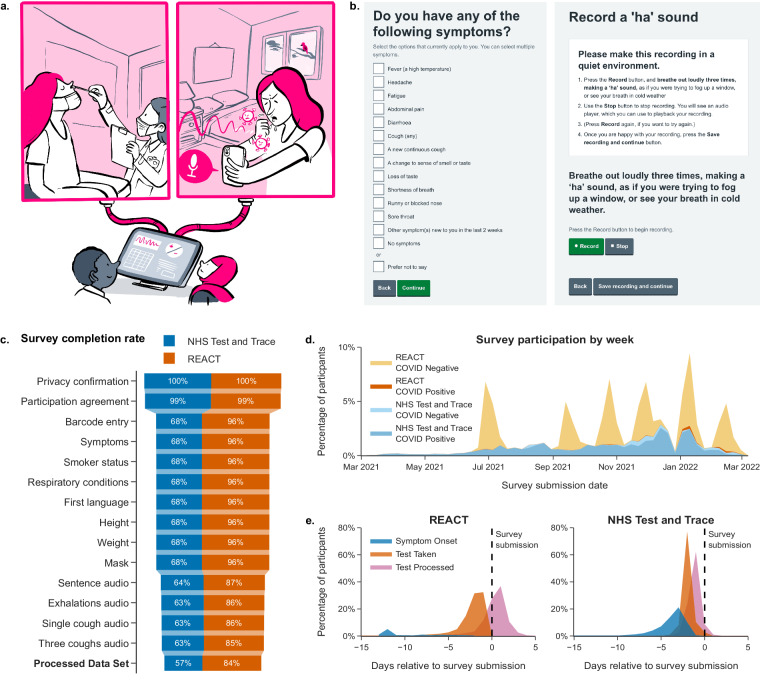
Study recruitment: (**a**) Illustration of dataset components. Participants of the REACT-1 study, or NHS Test and Trace patients underwent a test for SARS-CoV-2 infection. In this illustration the swab is healthcare worker-administered, however the majority of swab samples were self-administered in this study. Individuals from both cohorts were contacted and prompted to complete a digital survey, including recording a volitional cough and other respiratory sounds. SARS-CoV-2 test result data and associated information was combined with survey results and audio recordings, and was de-identified. This data descriptor document describes the process of producing the combined dataset, and its contents. This illustration is created by Scriberia with The Turing Way community (used under a CC-BY 4.0 licence 10.5281/zenodo.6821117). (**b**) Screenshots of the ‘Speak up and help beat COVID’ digital survey. (**c**) Participant survey completion rate by survey question across both recruitment modes. Completion counts were only collected for the ‘beta’ survey phase, described in Methods - Data Collection (with 59,431 participants equalling 81.4% of total dataset). (**d**) Participant records as a percentage of dataset total by week of survey submission, recruitment source, and SARS-CoV-2 test result. Individual REACT-1 survey rounds can be seen as peaks at irregular intervals. (**e**) Time of symptom onset, SARS-CoV-2 test swabbing (test start date), and SARS-CoV-2 test processing in relation to time of study survey submission in days, for each recruitment source. Percentages shown as the dataset total for each recruitment source. Symptom onset records shown only where symptoms were reported. Participants had been made aware of their SARS-CoV-2 test results on or shortly after the test processed date. REACT-1 participants who had an influenza test would have completed the test swab on the same date as their SARS-CoV-2 test, but would not have been made aware of the result.

UKHSA developed an online survey to collect a novel SARS-CoV-2 bioacoustics dataset (Fig. 1a,b) in England from 2021-03-01 to 2022-03-07 (Fig. 1d), during dominant transmission of the Alpha and Delta SARS-CoV-2 variants and some Omicron variant sublineages^7^. Participants were recruited after undergoing testing for SARS-CoV-2 infection as part of the national “*Real-time Assessment of Community Transmission”* (REACT-1) surveillance study (https://www.imperial.ac.uk/medicine/research-and-impact/groups/react-study/studies/the-react-1-programme/) and the NHS Test and Trace (T&T) symptomatic testing programme in the community (known as Pillar 2)^8^. To facilitate independent validation of existing models, audio samples common across existing studies were collected in the online survey, including: volitional (forced) cough, an exhalation sound, and speech. These were linked to SARS-CoV-2 testing data (method, results, date) for the test undertaken by the participant either as part of REACT-1 or T&T. Further data on participant demographics (age, gender, ethnicity, first language, location) and symptoms (type and date of onset) were collected in the online survey to monitor potential bias.

The UK COVID-19 Vocal Audio Dataset^9^ is designed for studies examining the possibility of classification of SARS-CoV-2 infection from vocal audio, including for the training and evaluation of machine learning models using PCR as a gold-standard reference test^10^. The inclusion of influenza status (for REACT-1 participants in REACT rounds 16–18) and symptom and respiratory condition metadata may provide additional uses for bioacoustics research.

All summary statistics described in this manuscript reflect the open access version of the UK COVID-19 Vocal Audio Dataset^9^ unless otherwise stated. Differences between the protected and open access dataset are described in Methods - Data Anonymisation. The protected version of the UK COVID-19 Vocal Audio Dataset is fully documented in our pre-print data descriptor^11^.

## Methods

### Survey design

Survey questions and responses are listed in Supplementary Table S1. Survey questions were designed to align to existing vocal acoustic data collection studies (see Table 1) and prevalence studies^12^, so that future comparisons of study demographics could be made if necessary. These include variables that could be captured in vocal audio acoustic features and/or could be confounded with SARS-CoV-2 infection status^13,14^, for example, respiratory symptoms, smoker status, and respiratory health conditions.

The participant’s testing provider collected data on age, gender, ethnicity, geographical area and SARS-CoV-2 test result (and associated information such as test type and PCR cycle threshold information, if available). To minimise data entry fatigue, these were linked to survey responses and not collected again through the survey.

Survey variables were also chosen to align with existing government surveys for ease of comparison: options available for ‘first language’ reflected those available in the ONS 2011 Census^15^; symptom options combined those available in the ONS Coronavirus Infection Survey^12^ and the NHS Test and Trace symptom self-screening tool prior to April 2022 (https://www.nhs.uk/conditions/covid-19/covid-19-symptoms-and-what-to-do/). Additional symptom options were added 2021-07-21 (‘other symptoms new to you in the last 2 weeks’) and 2021-08-11 (‘runny or blocked nose’, ‘sore throat’) to capture symptoms reported at a higher frequency in COVID-19 variants circulating at the time^16^. All questions allowed a ‘prefer not to say’ option to maintain participant control on the data they chose to share and to minimise non-response bias.

The final survey questions requested participants to record short audio segments using the microphone on their device, where the user interface for making the recordings was embedded in the online survey. Audio recordings, in order of participant submission, were: a sentence read aloud, three successive *“ha”* exhalation sounds, one volitional cough, and three successive volitional coughs. Audio prompts were chosen to be similar to those of existing datasets (see Table 1), so that models trained on other datasets could be independently evaluated with this dataset. On completion of the survey, responses including audio data were sent to a secure server and temporarily held before being sent to UKHSA. Screenshots of the survey are shown in Fig. 1b.

Two cough recordings were captured of one and three successive coughs, matching the prompts for cough recordings captured in previous studies (see Table 1). A cough is an innate reflex to remove irritation in the respiratory tract, in order to enhance gas exchange. Coughs are typically associated with respiratory infection, and a new, persistent cough was one of three ‘classic’ COVID-19 symptoms, however it was less prominent compared to other respiratory symptoms in later variants^16^. The difference between a reflexive and volitional cough should be noted, where a volitional cough may differ in duration and power^17^, and may be affected by the participants surroundings and emotional state. All coughs recorded in this study should be volitional, although a volitional cough may trigger a reflexive cough. Instructions were given to record the cough samples at an arm’s length, following the advice provided to participants of the COUGHVID study^18^, to reduce the risk of the audio recording being distorted (clipped). Participant instructions included guidance on coughing alone in a room or vehicle to reduce risk of COVID-19 transmission to others. Prompts and instructions are listed in Supplementary Table S1. The first (out of four total coughs) per participant may involve more fluid clearance. Of the successive (final three) coughs, the first was likely to be the most powerful, and successive coughs were likely to decrease in acoustic power as the participant had less time to inhale.

Exhalation sounds were also collected, as in previous studies. Breathing sounds are used in lung auscultation to identify narrowed airways or excessive fluid^19^ in the respiratory tract, although the clinical utility of external recordings (without a stethoscope) has not been established. Participants are prompted to record three short, powerful exhalations (*“ha”* sounds, as if the participants “were trying to fog up a window, or see their[sic] breath in cold weather.”). Participants were recommended to make this recording in a quiet environment to reduce background noise. For this recording, there was no direction around distance from participant to the recording device.

A sentence of speech, read from text, was also collected. Vowel sounds (such as ‘aah’ or ‘ee’) are used in lung auscultation (egophony) and to examine the vocal tract (such as contraction of the soft palate^20^). As speech is a combination of many varying vocal tract configurations over time, making it a more complex sample (anatomically) than coughing or breathing, it is more likely to be prone to biases in cognition, literacy, and accent and other learnt speech patterns. However, speech samples may potentially be more rich in acoustic features, particularly since smart device microphones, audio data processing, and the majority of vocal audio feature extraction models are configured for speech. Speech is produced through volitional manipulation of the vocal tract, where the shape of air cavities and air pressure is varied. A short sentence, *“I love nothing more than an afternoon cream tea”*, was chosen, combining several vowel and nasal sounds in a single recording.

### Recruitment

Participants were recruited through two existing SARS-CoV-2 infection testing pathways in parallel: (1) a community prevalence survey and (2) a government testing service. They were invited to take part in the study after they underwent testing. Survey responses and audio recordings were then linked to their test result. Inclusion criteria across both recruitment channels were: being 18 years of age or older and having a COVID-19 test barcode number. Participants were also advised to participate only if they had tested in the last 72 hours, although 13.2% of REACT-1-recruited and 2.1% of NHS Test and Trace-recruited participants in the dataset have a submission delay exceeding 72 hours, see Fig. 1e. (submission delay is described in the participant metadata file, see Supplementary Table S3). Participation was completely voluntary. This study includes some participants with a permanent address in the UK devolved administrations (Northern Ireland, Scotland, Wales), however, the data is disproportionately England-sampled due to the England-only recruitment of the majority of recruitment routes.

Participants were recruited via the Real-time Assessment of Community Transmission-1 (REACT-1) study. REACT-1 was commissioned by the UK Department of Health and Social Care to estimate the prevalence of SARS-CoV-2 infection in the community in England (and influenza A and B in later survey rounds). This was carried out by Imperial College London in partnership with Ipsos MORI using repeat, random, cross-sectional sampling of the population. Participants were randomly selected from National Health Service England records (which include almost the entire population) and sent a letter of invitation, with the aim of creating a representative sample of the population for each survey round (although actual response demographics vary, see Usage Notes). Participants were provided with instructions to take a throat and nasal self-swab and were asked to respond to an online/telephone survey about their demographics, symptoms and recent behaviours. The swab was either posted or collected by courier for PCR testing at laboratories. For rounds 13–18 (REACT survey dates from 2021-06-24 to 2022-03-01), participants were asked if they agreed to be contacted about further research led by the UKHSA. After sending their swab to a laboratory and completing the REACT-1 survey, those who agreed to be contacted were sent an email invitation to the online survey for this study which included audio recordings (survey questions and responses listed in Supplementary Table S1). 12.2% of the 295,493 individuals contacted for recruitment in REACT rounds 14–18 participated in the study and are included in the final dataset. Supplementary Table S2 lists participant cohorts and recruitment methods in further detail.

Participants were also recruited via SARS-CoV-2 testing services delivered by NHS Test and Trace (T&T). The purpose of this recruitment channel was to increase the number of survey responses linked to a positive PCR test result to better balance the combined dataset by SARS-CoV-2 infection status. Where the prevalence of SARS-CoV-2 infection of the REACT-1 cohort was expected to be similar to the prevalence in the general population, a higher proportion of positive cases may be needed for the development of SARS-CoV-2 infection status classification models. During the study period, people were advised to seek a PCR-test through T&T (swab testing for the wider population, as set out in government guidance, known as Pillar 2^8^) if they were (i) experiencing COVID-19 symptoms, (ii) identified as a close contact of a positive case, or (iii) taking a confirmatory PCR test following a positive rapid antigen (lateral flow) test (until 11th January 2022). Tests were free to use and available at test sites or for home delivery. Throat and nasal swabs were mostly self-administered at test sites or in participants’ homes, before being sent to laboratory sites for testing^8^.

A subset of participants recruited through T&T reported lateral flow test results. Lateral flow testing of SARS-CoV-2 antigen was open and free to the public in the UK, including for asymptomatic testing, and in the majority of cases was performed by the participant and reported through the NHS COVID-19 app or website.

Those that underwent testing could opt-in to be contacted about participating in research. An eligible subset of these were then contacted by text, email or phone call to invite them to participate in the study. Supplementary Table S2 lists participant cohorts and recruitment methods in further detail. Eligible populations were defined by SARS-CoV-2 infection and symptom status over a distribution of ages. Recruitment was initially focused on those receiving a positive test result. Between 2021-11-11 and 2022-03-04 recruitment was targeted at 50% of a random sample of all that includes those testing positive, negative or with a void PCR test result. Participants were linked to an online survey where prompts and audio recording format were uniform across recruitment channels.

A small proportion of participants prior to 2021-03-17 were recruited via information leaflets at regional COVID-19 test sites, displaying a QR code linked to the study survey.

Participants were also recruited from The ONS Coronavirus Infection Survey (https://www.ons.gov.uk/surveys/informationforhouseholdsandindividuals/householdandindividualsurveys/covid19infectionsurveycis) and the COVID-19 Challenge study (COV-CHIM 01, https://www.hra.nhs.uk/planning-and-improving-research/application-summaries/research-summaries/cov-chim01-sars-cov-2-dose-finding-infection-study_v10/) however lower participant counts from these recruitment methods could not guarantee participant anonymisation and so these participants are not included in the UK COVID-19 Vocal Audio Dataset.

### Data collection

The online survey ‘Speak up and help beat coronavirus’ (https://www.gov.uk/government/news/speak-up-and-help-beat-coronavirus-covid-19) was accessible via compatible internet connected devices with the ability to capture audio recordings, such as smartphones, tablets, laptops, and desktop computers. Participants recruited from T&T testing services were contacted to take part in the study after completing a SARS-CoV-2 test and agreeing to take part in research (see Supplementary Table S2 for modes of contact). Those recruited from the REACT-1 cohort were sent an email invitation. Participants reviewed the participant information and confirmed their informed consent to take part. An automated check to confirm a participant’s device was able to record audio was integrated into the digital survey, which participants needed to complete before continuing. Participants accepted a participation agreement and privacy statement outlining how their survey and test data would be linked, how their data would be used for research, and made available for reuse by researchers. Next, they entered their test/personal barcode number, followed by responses to questions about their demographics, comorbidities and any symptoms they were currently experiencing. Participants responded to survey questions from a choice of predefined responses (survey questions and multiple-choice responses listed in Supplementary Table S1, survey completion rates listed in Fig. 1c).

Until 2021-08-12, the ‘alpha phase’ gathering solution was hosted at www.ciab2021.uk (used by 18.6% of participants, noted as ‘alpha’ in the ‘survey_phase’ metadata variable, see Supplementary Table S3), and from 2021-08-13 to 2022-03-07, the ‘beta phase’ data gathering solution was hosted at www.speakuptobeatcovid.uk (used by 81.4% of participants, noted as ‘beta’ in the ‘survey_phase’ metadata variable, see Supplementary Table S3). To ensure robustness, both data gathering solutions were tested extensively to ensure data gathered was recorded accurately in the databases of the respective solution, and to confirm that the data was subsequently transferred to UKHSA correctly. This included end-to-end tests with dummy submissions.

The API and associated configuration used for recording audio in the ‘alpha’ solution was replicated as-is in the ‘beta’ solution. Recordings through both data gathering solutions were compared to check consistency, including comparison of Fast Fourier Transform (FFT) spectra, file format, and sampling rates using the python librosa library (https://github.com/librosa/librosa). The solution delivery teams for both ‘alpha’ and ‘beta’ data gathering solutions confirmed that no post-processing of the stored audio files occurred for either solution.

### Data linking

A data pipeline was designed to merge the primary data gathered in support of this study (the submission data) with the secondary data (the SARS-CoV-2 test results data) gathered by each testing provider. Data pipeline code was drafted and peer-reviewed by the UKHSA study team, and was also reviewed independently by the data wrangling team from The Turing-RSS Health Data Lab. Survey data and audio recordings submitted by the participant were linked to the SARS-CoV-2 test result data (date, result, test type, testing laboratory, PCR cycle threshold values (if provided), and estimated viral load (if provided)) for the test they underwent prior to being recruited. They were also linked to demographic information of potential additional utility to the dataset (age, gender, ethnicity, geographical information, COVID-19 vaccination status) which was collected by the testing provider.

Test barcodes were used to link T&T data to survey data. Test results data from T&T-recruited participants were sourced from the National Pathology Exchange (NPEx) database that stored test result data from across the T&T laboratory network and home-based lateral flow test results. Participant age, gender, ethnicity, and location were derived from data entered by the participant when registering for a test and stored in the NPEx database.

The study team generated a set of unique personal codes, which were provided securely to Ipsos MORI, who included a code in each email invitation to participate in this study. These personal codes differed in format from T&T barcodes to avoid accidental duplication. This personal code was used to link REACT-1 data to survey data. For REACT-1-recruited participants, the test result and associated data were provided by Ipsos MORI as a filtered extract of the REACT-1 study data including only the records and fields relevant to this study. Participant codes were extracted from responses to the survey for this study and transferred via approved protocols to Ipsos MORI. Ipsos MORI checked for duplicate entries before then extracting and sharing the test result data for UKHSA to link back to survey submissions.

The pipeline script was designed to exclude any participant submissions from the final dataset that could not be linked to valid test results data using the test identifier code submitted by the participant. The test identifier codes were not publicly available and were provided to the participant through the relevant recruitment route, mitigating the risk of any submissions where the primary data gathered was provided by a different individual from the secondary test results data.

To further mitigate this risk, as well as provide a metric required for the study exclusion criteria, the pipeline script calculates the delay between the time of the participant’s submission to the primary data gathering solution, and either the swab time or lab processing time for the SARS-CoV-2 test the participant conducted. This delay was calculated as the difference between the time of the participant’s submission to the primary data gathering solution, and either the swab time or lab processing time for the test ('submission_delay' variable, see Supplementary Table S3). This variable enables results to be filtered out from the study data if there was a significant delay between submission and SARS-CoV-2 test, as this could either suggest the participant entered the test identifier incorrectly and has been associated with the wrong test result record, or due to the delay the test may no longer be indicative of the participant’s SARS-CoV-2 infection status.

Participant submissions which could not be linked to a valid SARS-CoV-2 test result were excluded from the final dataset. Test barcode numbers were removed at the end of the study, and replaced with a random identifier associated with an individual participant ('participant_identifier' metadata variable, see Supplementary Tables S3, S4), de-linking the participant metadata from their test barcode number and identifiable information associated with it.

### Data cleaning

Duplicate entries from the same participant were removed where possible so that the data tables have one row (equivalent to one survey entry) per participant. For T&T-recruited participants, repeat individuals were identified in the source test results data table, and repeat submissions were removed keeping only each individual’s first submission, which would be closer to the time of the participant’s SARS-CoV-2 test. For the REACT-1-recruited participants, Ipsos MORI indicated which submissions related to individuals who had previously taken part in the study, and repeat submissions were removed keeping only first submissions. There remains a residual risk that some individuals took part in both recruitment groups and as a result have made multiple submissions in the study data, however, this is expected to be a low volume due to the national scale of both recruitment approaches. The removal of duplicates cannot be guaranteed prior to 2021-06-01 (1.4% of participants), as a shorter agreed personal data retention period for this ‘pilot’ phase of data collection meant that test barcodes could not be stored for the duration of the study.

Variable categories were standardised for uniformity across recruitment channels where there was overlap between categories. REACT category names were typically renamed to match T&T category names. Data types were standardised by variable (unless disclosure controls required mixed data types). List variables from survey multiple choice questions were one-hot encoded.

### Data anonymisation

To enable wider accessibility, an open access version of the UK COVID-19 Vocal Audio Dataset^9^ was produced to protect participant anonymity according to the ISB1523: Anonymisation Standard for Publishing Health and Social Care Data standards (https://digital.nhs.uk/data-and-information/information-standards/information-standards-and-data-collections-including-extractions/publications-and-notifications/standards-and-collections/isb1523-anonymisation-standard-for-publishing-health-and-social-care-data). The audio recordings of read sentences were removed in the open access version of the dataset, on the basis that non-distorted speech data can constitute sensitive biometric personal information, carrying a risk of participant reidentification. Additionally, several participant metadata variables were either removed, binned, obfuscated, or pseudonymised to meet the requirement of K-3 anonymity after combining all variables relating to personal data. The total number of participants remained unchanged.

Specifically, audio metadata relating to the audio recordings of read sentences were removed, including audio transcripts. Participant metadata variables relating to ethnicity, first language, vaccination status, height, weight, and COVID-19 test laboratory code were removed. Participant age was binned into age groups, and survey recruitment source variables were binned into general recruitment source groups. All dates were indexed to a random date and obfuscated with ± 10 days random noise. All dates included in the metadata are indexed to the same random date for comparison, and all dates for each participant have the same level of noise applied, to allow for the calculation of time differences at the participant level. Geographical information was originally collected at the local authority (sub-regional administrative division) level, and was later aggregated to region (first level of national sub-division) level and pseudonymised to avoid the risk of participant disclosure.

A flagged COVID-19 test laboratory code in the protected dataset indicated a laboratory with reported false COVID-19 test results. All results from this laboratory have been set to None for the open dataset version, slightly altering overall counts of positive, negative and invalid results. All summary statistics presented in this article reflect the open access version of the UK COVID-19 Vocal Audio Dataset^9^ unless otherwise stated. A data dictionary for the open access version of the UK COVID-19 Vocal Audio Dataset metadata is provided in Supplementary Tables S3, S4.

### Ethics

This study has been approved by The National Statistician’s Data Ethics Advisory Committee (reference NSDEC(21)01) and the Cambridge South NHS Research Ethics Committee (reference 21/EE/0036) and Nottingham NHS Research Ethics Committee (reference 21/EM/0067).

## Data Records

The open access version of the UK COVID-19 Vocal Audio Dataset has been deposited in a Zenodo repository (10.5281/zenodo.10043977)^9^, and is available under an Open Government License (v3.0, https://www.nationalarchives.gov.uk/doc/open-government-licence/version/3/). Additional data records as part of the protected dataset version may be requested from UKHSA (DataAccess@ukhsa.gov.uk), and will be granted subject to approval and a data sharing contract. To learn about how to apply for UKHSA data, visit: https://www.gov.uk/government/publications/accessing-ukhsa-protected-data/accessing-ukhsa-protected-data.

There were 72,999 participants included in the final dataset, with one submission per participant. This included 25,706 participants linked to a positive SARS-CoV-2 test. The majority of these submissions (70,565, 96.7%) were linked to results derived from PCR tests (RT-PCR, q-PCR, ePCR), followed by lateral flow tests (1,925, 2.6%) and LAMP (loop‐mediated isothermal amplification) tests (244, 0.3%). Of all test results, 257 (0.4%) were inconclusive with an unknown or void result. This dataset represents the largest collection of PCR-referenced audio recordings for SARS-CoV-2 infection to date, with approximately 2.6 times more participants with PCR-referenced audio recordings than the Tos COVID-19 dataset (with 27,101)^21^.

**Fig. 2:**
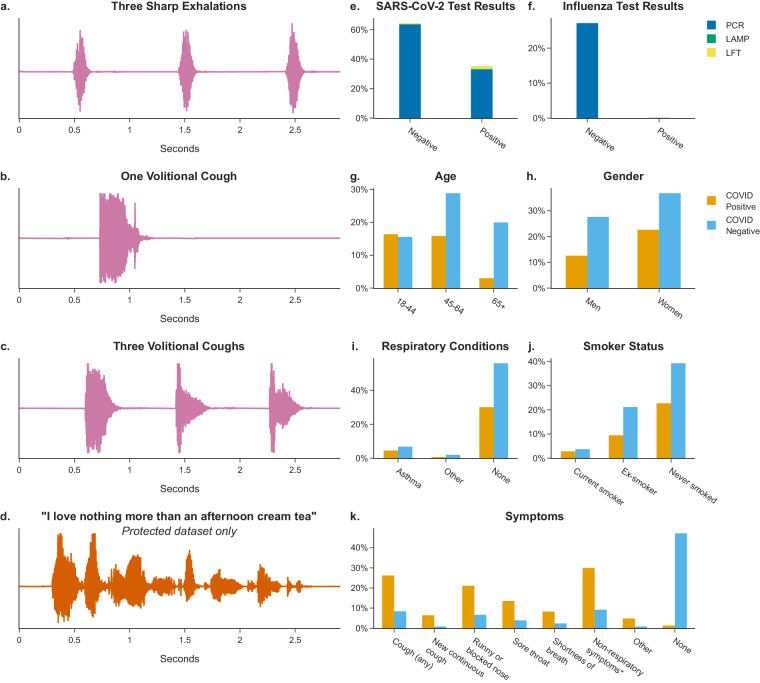
Dataset summary: All percentages from the dataset total (72,999 participants). ‘Prefer not to say’ or missing responses are not visualised (variable completeness statistics are listed in Supplementary Tables S3, S4). Multiple categories with low counts grouped for visualisation. (**a**–**d**) Waveforms of the four audio recordings captured for each participant (three successive exhalations, one volitional cough, three successive volitional coughs, a read sentence; here recorded as an example by the primary author at 44.1 kHz when asymptomatic and with unknown SARS-CoV-2 infection status). (**e**) Percentage of total participants by COVID-19 status and test type. (**f**) Percentage of total participants by Influenza status (A or B) and test type. (**g**) Participant age group by SARS-CoV-2 test result. (**h**) Percentage of total participants by gender (REACT-1/T&T categories) and SARS-CoV-2 test result. (**i**) Percentage of total participants by self-reported respiratory condition and SARS-CoV-2 test result. (**j**) Percentage of total participants by smoker status and SARS-CoV-2 test result. (**k**) Percentage of total participants by self-reported symptoms and SARS-CoV-2 test result. *Non-respiratory symptoms include fever or high temperature, headache, abdominal pain, loss of taste, and changes to sense of smell or taste.

The majority (44,565 participants, equalling 61.0% of total dataset) of participants were recruited via REACT-1. The remaining 28,434 participants, (equalling 39.0% of the total dataset) were recruited via T&T testing services. Figure 2 shows a summary of participant attributes. The median age of participants was 53 years old and 59.6% of participants were female (43,537 participants). Participants with a positive SARS-CoV-2 test result were more likely to report that they were experiencing respiratory symptoms (90.7% of participants testing positive reported respiratory symptoms vs. 20.9% of participants testing negative reported respiratory symptoms). This dataset has additional potential uses for bioacoustics research, as 9,749 (13.4%) participants reported a pre-existing respiratory health condition (comorbidity) of which 8,249 (11.3%) reported asthma. Participants recruited from REACT rounds 16–18 (19,859 participants, 27.2% total) have linked influenza PCR test results, with 33 participants testing positive for influenza A and 28 testing positive for influenza B. Several recruitment biases were apparent and are discussed in Usage Notes.

**Fig. 3:**
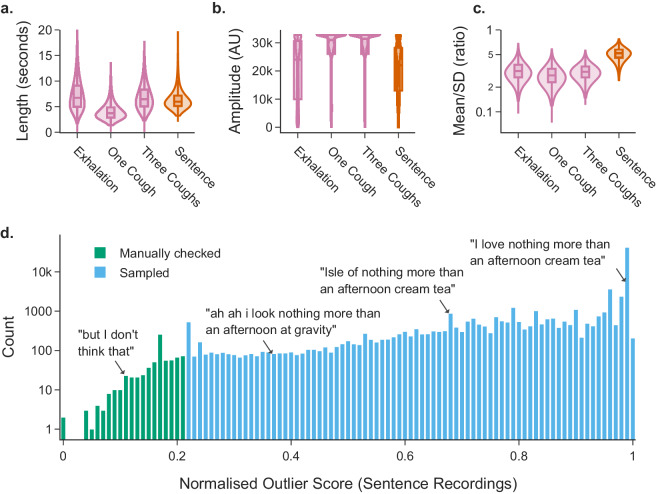
Audio data technical validation: (**a**) The distribution of audio clip length in seconds for each audio modality. (**b**) The distribution of audio clip amplitude (difference between maximum and minimum of absolute signal) for each audio modality. (**c**) The distribution of audio clip absolute signal means divided by absolute signal standard deviation for each audio modality; (**d**) Normalised anomaly scores of sentence audio clip transcript embeddings, where the 1000 most anomalous were checked manually for personal information. Sentence audio recordings and transcripts are included in the protected dataset version only.

Audio was recorded in *.wav* format (86.2% of submissions had a sample rate for all recordings of 48 kHz, 13.2% of submissions had a sample rate for all recordings of 44.1 kHz) and had a maximum length of 64 seconds (see Fig. 3a). Three audio files (one for each recording) are provided for each of the 72,999 participants (unless missing, see Technical Validation). The protected version of the dataset contains one extra audio file (of recorded speech) per participant with a maximum length of 72 seconds.

Metadata, including audio filenames, are provided in .csv files, linked by a participant identifier code. Metadata data dictionaries are provided as tables for participant metadata (Supplementary Table S3) and audio metadata (Supplementary Table S4).

Additional data available include the participant training/testing splits for the investigations reported by *Coppock et al*.^10^ (in both open access and protected versions of the dataset) and OpenSmile features^6^ generated from the audio files (in the protected dataset only).

## Technical Validation

Audio *.wav* files were parsed and metadata extracted including sample rate, number of samples, and number of channels using the python scipy library (https://github.com/scipy/scipy). Using the audio data and extracted metadata, the audio length in seconds, audio amplitude (absolute maximum - absolute minimum signal), and audio signal-to-noise ratio were calculated for each file. Figure 3a–c show the distribution of audio file length, audio amplitude, and audio absolute signal mean to standard deviation ratio for each audio recording type, respectively. All files had one audio channel. Audio metadata is available in the audio_metadata *.csv* file of the dataset. 2.5% participants were missing one or more audio files, or had audio files with a size of <45 bytes, and were flagged with the missing_audio variable. Empty audio files are not included in the open access dataset, and so a small number of flagged audio file paths in the audio_metadata table will list a non-existent file. Audio metadata variable completeness by recording type is included in Supplementary Table S4.

Audio files were screened systematically to reduce the risk of disclosure of personal information. This could arise where participants had failed to follow the study instructions and the audio prompts, instead accidentally or intentionally disclosing personal information such as their name. An analytical pipeline was developed to identify outliers from the total 289,696 audio files (217,162 of which are in the open access dataset), where the outliers were screened manually. A speech-to-text model (fairseq S2T, small version, pre-trained weights available at https://huggingface.co/facebook/s2t-small-librispeech-asr)^22^ was run on audio files, producing a text transcript. A text-to-embedding model (MPNet Transformer v2, pre-trained weights available at https://huggingface.co/sentence-transformers/all-mpnet-base-v2)^23^ was run on the transcript, producing a format of the transcript (an embedding) that was quantified and compared with the prompt to identify outliers, or speech that differs from the prompt.

Each embedding was then ranked by its similarity to the prompt using a Support Vector Machine (SVM) model. The 1000 sentences which differed most were then inspected manually to check for disclosure of personal information, and non-outlier files were randomly sampled. Figure 3d shows the distribution of the similarity rank for every audio file for the sentence modality. The majority of transcripts (56.1%) show the correct sentence (*“I love nothing more than an afternoon cream tea”*). Most others sampled show a similar sentence, misinterpreted by the speech-to-text model, with 95.6% of transcripts containing the substring *“nothing more”*, and 91.8% containing *“nothing more”* and *“afternoon”*, with the other words commonly mis-transcribed. A small proportion of sampled outlier transcripts show alternative speech from the participant. These transcripts and associated audio files were retained unless personal information was disclosed (one audio file was found to contain personal information and was truncated). Other outliers had media playing in the background, or others show artefacts of noise e.g. “*of of of of of of of of of…*”. Sentence transcripts and outlier scores are available in the audio_metadata table of the protected version of the dataset.

Several data filtering steps are recommended when using this dataset for the development of models with the intention of SARS-CoV-2 infection classification, including filtering to include only participants with a PCR-type SARS-CoV-2 test, participants whose test was not carried out in a laboratory with reported testing errors, and participants who completed the study survey within a defined delay (e.g. 10 days) of their SARS-CoV-2 test. *Pigoli et al*. describe these suggested data filtering steps in further detail^24^.

## Usage Notes

### The use of this dataset for SARS-CoV-2 infection status classification

For effective use of this dataset, users should be aware of limitations in the use of surrogate indicators such as vocal biomarkers in the development of SARS-CoV-2 infection status classification models. Many SARS-CoV-2 infections are asymptomatic, and the presentation of any symptoms may be dependent on the stage of infection, which may not necessarily correlate with viral load and transmissibility^25^. 9.3% of participants with a positive SARS-CoV-2 test result do not report any respiratory symptoms, and 3.8% report not having symptoms of any type. 20.9% of participants with a negative test result reported respiratory symptoms. The specificity of audio-based SARS-CoV-2 detection may also be dependent on the prevalence of other circulating respiratory viruses, which may have similar respiratory symptoms and effect on vocal audio. All participants with a positive test result for influenza also had a negative test result for SARS-CoV-2 infection. Other respiratory infections that may have a similar symptom profile were not tested in this study. Other recorded variables such as respiratory conditions and smoker status may be a confounding variable in the analysis of vocal biomarkers. 14.6% of participants with a positive SARS-CoV-2 test result also reported a respiratory health condition (including asthma, COPD, and emphysema). *Coppock et al*. further analyse the potential confounding effect of several variables in this dataset on SARS-CoV-2 infection classification^10^.

There is some selection bias in the recruitment for this study, where the majority of participants recruited via the REACT-1 surveillance study were SARS-CoV-2 negative at the time of participation, and the majority of the participants recruited via T&T were SARS-CoV-2 positive at the time of participation. This selection was necessary to produce a dataset with a relative balance of SARS-CoV-2 infection status, but researchers should note the varying composition of each recruitment population, which could be confounded with infection status. Additionally, within T&T recruitment, the recruitment method of some positive and negative cases varied (see Supplementary Table S2). *Pigoli et al*. document the potential confounding variables in this dataset due to recruitment^24^. Of particular note is symptom presentation, where participants recruited via T&T would have sought a PCR test due to having a positive lateral flow test (between 2021-03-29^26^ and 2022-01-11^27^) or having symptoms (at least one of: a high temperature; a new continuous cough; a change to sense of smell or taste) as per UKHSA guidance at the time of data collection^8^. Changes in UK testing policy, such as local surge testing or school and workplace testing policies would also create selection biases for the T&T population that varied over time. Compared to T&T, COVID-19 positive participants recruited via the REACT-1 study were less likely to be symptomatic. The distribution of symptom status was more likely to reflect that in the general population and be stable over time (in relation to SARS-CoV-2 prevalence). Users should note that not all those who were contacted to participate in the REACT-1 survey participated, creating some self-selection biases. Only those who participated in the REACT-1 survey were contacted to participate voluntarily in this study, leading to further self-selection biases.

*Coppock et al*. list seven core issues with existing COVID-19 audio research and the datasets used^28^. While we have designed this dataset attempting to address these issues; including providing PCR-confirmed infection status, providing demographic and health metadata for each participant, ensuring only one submission per participant, and publishing this dataset; several issues remain. Participants testing positive for SARS-CoV-2 infection at the time of participation may be aware of their infection status, particularly those recruited via T&T (Fig. 1e). This may introduce undocumented confounders in the audio recordings, such as behaviour when recording, the environment in which recordings are made, and participant emotions. Recruitment biases, discussed above, may also present undocumented confounders present in the audio data. Although there is little variation in audio sample rate across the dataset (see Data Records), we did not record device type, microphone hardware specifications, browser, or device operating system, which may have some effect on audio quality.

Although PCR is the gold-standard for detection of SARS-CoV-2 infection, users should note that it may be an imperfect label in categorising participants as infectious. Due to the amplification step, viral RNA can remain detectable by PCR long after live SARS-CoV-2 can be cultured from patient samples. Stratifying model evaluation by estimated viral load may remedy this, where studies have shown that the viral load threshold for transmission is ~1,000,000 viral RNA copies/ml^25^. We include viral load data where available (14.6% of submissions). Users are encouraged to use the covid_viral_load_category variable rather than the covid_viral_load, covid_ct_value or covid_ct_mean variables, as there is variation between tests including different gene targets (documented by covid_ct_gene). False negative PCR results are also possible, likely related to sampling technique, volume of fluid, and viral load. A meta-analysis found a pooled estimate of 94% PCR sensitivity^29^. PCR results from a laboratory reported to have made substantial testing errors have been made void in this dataset.

The period of data collection saw different SARS-CoV-2 variants (notably Alpha, Delta and Omicron) circulating in the UK, which have been reported to cause differing prevalence of symptoms to each other and to previous variants^16^. Dataset authors recommend against using the covid_ct_gene metadata variable to estimate SARS-CoV-2 variant causing infection (e.g. through S-gene dropout^30^), as this variable reports only a single gene target with the lowest cycle threshold value, and not all laboratories test for all genes.

Due to recruitment constraints, we were unable to include longitudinal data (multiple data entries by the same participant over time) for any participants, as is present in other datasets such as the COVID-19 Sounds^31^ dataset. As a result, this dataset is insufficient to study potential vocal changes throughout SARS-CoV-2 infection in the same individual. Temporal changes may be studied in a cross-sectional manner with appropriate controls using the symptom_onset variable.

Alternative COVID-19 and influenza-related uses of the dataset may include the development of generic respiratory symptom detection or cough and cough frequency detection, which may have utility in syndromic surveillance (if used in a privacy-preserving manner) or the monitoring of chronic disease in addition to acute disease. Any developed solution should be first trialled in the context of its application to provide evidence of patient safety, generalisability, and reported effectiveness.

### The use of this dataset for asthma status classification

Of the 72,816 participants responding to the survey prompt regarding existing respiratory conditions, 8,249 (11.3%) report having asthma. This provides a large vocal audio dataset labelled with participant asthma status, which may be used for training or evaluating machine learning models for asthma status classification. While asthma is a condition characterised by respiratory symptoms, vocal audio should be considered a surrogate indicator compared to established diagnostic methods such as those measuring expiratory flow or inflammation^32^. Unlike SARS-CoV-2 and influenza infection status, which is confirmed with a diagnostic test in this dataset, asthma status is self-reported. This can introduce confirmation bias, where some undiagnosed asthma participants may be labelled as not having asthma. SARS-CoV-2 infection status and other respiratory conditions may be confounding factors in the use of vocal audio for asthma status classification. 39.5% of participants reporting asthma also have a linked positive SARS-CoV-2 test result, 0.1% have a linked positive influenza A or B test result, and 4.8% report another respiratory condition including COPD and emphysema.

### Dataset demographic biases

**Fig. 4:**
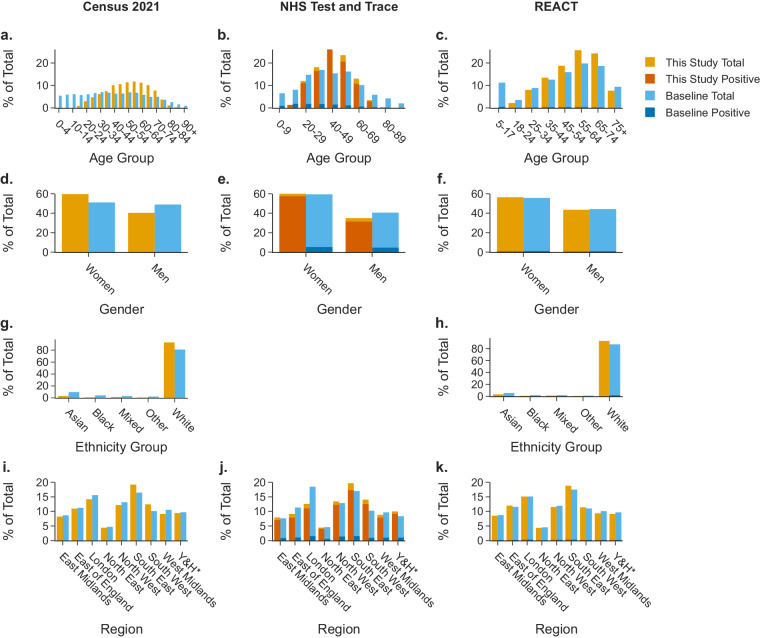
Demographic biases in the dataset: Distribution (**a**–**c**) of age groups (note referenced studies use different groupings for age), (**d**–**f**) genders, (**g,h**) ethnicity groups, and (**i**–**k**) UK regions in the study dataset in comparison to general population (Census 2021)^46^, NHS Test and Trace users who took a PCR or LAMP test 25-02-2021 to 02-03-2022^33^, participants in REACT-1 rounds 13–18^34–37^. Data for England only. Where study data demographic distributions are compared to NHS Test and Trace and REACT-1, only the study data subset from each respective recruitment channel are used. NHS Test and Trace and REACT-1 data subset plots also compare the split of SARS-CoV-2 positive and negative cases between this study and the cohort reference. Category groups are created according to how baseline data is reported. ‘Unknown’ categories are not displayed. NHS Test and Trace ethnicity data by week is not publicly available. *Y&H = Yorkshire and The Humber. Granular age data, ethnicity group and UK region data are available only in the protected version of the dataset, and are presented here for context.

Demographic imbalances are present within the dataset, where study participants were more likely to be White British, women, and aged 35–74 years than the general UK population. Figure 4 compares the distribution of ages, genders, ethnicities, and region of habitation of study participants in comparison to the general population (as recorded by the 2021 UK Census), patients using T&T in the weeks of data collection^33^ (compared to only T&T-recruited study participants), and REACT-1 study participants for the relevant study rounds^34–37^ (compared to only REACT-1-recruited study participants). Granular age data, ethnicity group and UK region data are available only in the protected version of the dataset, and are presented here for context. Some dataset biases can be seen to be partly inherited from the two recruitment channels, as in the case of gender, where more patients or participants were women in comparison to the general population. Other biases, such as age biases, can be seen to be exacerbated by the recruitment of this study, where fewer participants over the age of 80 years were seen in the two recruitment channels (and none under the age of 18 years, due to study exclusion criteria). The survey for this study was only made available in English which could have exacerbated language and ethnicity biases. Similar demographic biases were present in other voluntary digital surveys for COVID-19 research and surveillance in the UK, such as the COVID Symptom Study^38^. The nature of study recruitment and participation may exclude certain demographic groups with limited digital literacy or access to digital infrastructure^39^. The voluntary nature of this study may exclude certain demographic groups with limited available time due to employment and/or care commitments^40^. We recommend that researchers using this dataset to train audio classification models should report test accuracy statistics stratified by demographic variables to communicate any model biases.

The substantial majority of participants (94.5%) report English as their first language or the language most commonly spoken at home (if they have two or more first languages). Therefore, any analysis of speech data may only be valid in English speakers and should be tested in other populations before language-generalisable results are reported. Regional accents may have an effect on speech models. Recruitment is relatively balanced by administrative region, particularly for REACT-1-recruited participants. As a result, the audio data may contain a representative sample of regional English accents. Most study participants were recruited in England and so, more speech data would be needed to evaluate accents which are more common outside of England.

The participant metadata variables not captured directly in the digital survey (digital survey questions and related variables listed in Supplementary Table S1) were shared by the relevant recruitment channel (see Methods - Survey Design), where format and prompt vary. Efforts have been made to standardise data format between recruitment channels and are listed in the participant metadata dictionary (Supplementary Table S3). Users should note that some calculated variables, such as symptom_onset, continue to have values of distinct distributions despite this standardisation due to the variation in recruitment methods (patients seeking a test vs survey population). T&T- and REACT-derived demographic variables had limited multiple-choice options and limited ethnicity and gender categories were available, meaning some demographic analyses are not possible.

### Supplementary information

Survey Questions and Response Options

Granular Recruitment Information

Participant Metadata Dictionary

Audio Metadata Dictionary

## Data Availability

Summary statistics and relevant figures and can be reproduced from the open access or protected versions of the UK COVID-19 Vocal Audio Dataset using code found here: https://github.com/alan-turing-institute/Turing-RSS-Health-Data-Lab-Biomedical-Acoustic-Markers/ which is archived^41^ under 10.5281/zenodo.11208315.
